# Gynæcology of the Seventeenth Century

**Published:** 1880-04

**Authors:** James I. Tucker

**Affiliations:** Chicago


### ARTICLE XII.

*Editors* CHICAGO MEDICAL JOURNAL AND EXAMINER:

In my article on the gynæcology of the seventeenth century, I said in conclusion that one would be led to think the Dr. Courrade must have known and used the speculum because he described so accurately granulations, ulcerations, etc., of the collum uteri. But I certainly did not mean to convey the impression that the speculum was unknown up to that time, and consequently a product of much more recent inventive genius. I meant only that it was eclipsed in the general darkness of mediæval times. The speculum (*speculum ani et vaginæ uteri*) was known long before the Christian era. Ætius, who flourished in the sixth century A. D., and Paul of Ægina, his successor, describe the instrument and its uses distinctly, and specula have been unearthed from various ruins, including a bivalve, which is now preserved in the Museo Borbonico.

"Probably all wit and all wisdom have often been fully explored and again quite forgotten," said Aristotle two thousand years ago, and it is doubtless true.

It is to be regretted that so little attention is given now-a-days to the history of medicine. It is possible that our pride might sometimes be mortified in the presence of facts, which were worn out and thread-bare when Arabians, Egyptians, Greeks and Romans were in their glory, and which to the modern world are, to say the least, not fully understood.

Notwithstanding what I have said above about the speculum as being a product of classic genius and an important factor in the present *renaissance*, it is nevertheless my firm conviction that, with the evolution of the sense of touch, the speculum, except for strictly surgical purposes, will be regarded as a relic of barbarism, and that he will be a poor diagnostician who will not be able to come to a correct conclusion concerning os-uterine and vaginal conditions by the aid of the sense of touch alone. In fact, I think we might now save a good deal of physical, to say nothing of moral torture, by trusting more to digital, which, in the light of modern psychology, means mental impressions.

CHICAGO, March 3, 1880. DR. JAMES I. TUCKER.

